# Association of dietary inflammatory index with mortality risk: a prospective analysis of the Korea National Health and Nutrition Examination Survey

**DOI:** 10.4178/epih.e2025017

**Published:** 2025-04-09

**Authors:** Dahyun Park, Hee Ju Jun, Garam Jo, Soyoung Kwak, Min-Jeong Shin

**Affiliations:** 1 Multidisciplinary Research Center for Public Health in Complex System, Korea University, Seoul, Korea; 2 Interdisciplinary Program in Precision Public Health, Graduate School of Korea University, Seoul, Korea; 3 Institute for BioMaterials, Korea University, Seoul, Korea; 4 Perlmutter Cancer Center, NYU Langone Health, New York, NY, USA; 5 Department of Population Health, NYU Grossman School of Medicine, New York, NY, USA; 6 Department of Biosystems and Biomedical Sciences, Korea University College of Health Science, Seoul, Korea

**Keywords:** Diet, Inflammation, Mortality, Cardiovascular diseases, Cancer

## Abstract

**OBJECTIVES:**

The energy-adjusted dietary inflammatory index (E-DII), a tool developed based on comprehensive research and literature reviews, is used to assess the inflammatory potential of specific diets. Although previous research has demonstrated an association between E-DII and mortality, longitudinal studies investigating a causal relationship in Asian populations are lacking. This study aimed to explore the prospective association between E-DII and the risk of all-cause, cancer, and cardiovascular disease (CVD) mortality using a population-based Korean cohort.

**METHODS:**

The analysis included data from 40,596 individuals who participated in the Korea National Health and Nutrition Examination Survey between 2007 and 2015. The exclusion criteria encompassed the diagnosis of cancer or CVD at baseline, pregnancy at baseline, and death within the first 2 years after baseline. The E-DII was calculated using data from 24-hour dietary recall interviews. Cox proportional hazard regression models were employed to calculate hazard ratios (HRs) with 95% confidence intervals (CIs) for mortality risk across E-DII tertiles.

**RESULTS:**

Over an 8.2-year follow-up period, 2,070 deaths were recorded. Compared with the lowest E-DII, a higher index was associated with an increased risk of mortality from all causes (HR, 1.45; 95% CI, 1.25 to 1.69), cancer (HR, 1.41; 95% CI, 1.09 to 1.81), and CVD (HR, 1.53; 95% CI, 1.07 to 2.18). The association between E-DII and all-cause mortality was particularly pronounced among individuals with metabolic conditions.

**CONCLUSIONS:**

Our findings suggest a strong positive association between high E-DII and increased mortality in Korean adults, especially those with metabolic disorders.

## GRAPHICAL ABSTRACT

[Fig f3-epih-47-e2025017]

## Key Message

This prospective cohort study analyzed nationally representative data from the Korea National Health and Nutrition Examination Survey (2007-2015) to examine the association between the energy-adjusted Dietary Inflammatory Index (E-DII) and mortality risk. A higher E-DII was significantly associated with increased risks of all-cause (HR, 1.45; 95% CI, 1.25-1.69), cancer mortality (HR, 1.41; 95% CI, 1.09-1.81), and cardiovascular mortality (HR, 1.53; 95% CI, 1.07-2.18), particularly among individuals with metabolic disorders.

## INTRODUCTION

Inflammation is a physiological response that plays a crucial role in defending against pathogens and promoting tissue repair [1]. In contrast, chronic inflammation and elevated serum levels of inflammatory markers have been linked to the development of various diseases, including cardiovascular disease (CVD), cancer, diabetes mellitus, and neurodegenerative disorders [2-4]. Notably, metabolic disorders pose a serious global health challenge, prompting considerable scientific interest in exploring anti-inflammatory strategies to mitigate their impact.

Extensive evidence supports the association between inflammation and lifestyle factors, especially dietary intake [5,6]. Numerous food components contribute to the inflammatory response, and the cumulative effect of interacting dietary factors may impact the severity of inflammation and related health outcomes [7,8]. The energy-adjusted dietary inflammatory index (E-DII) [9] is a recently developed comprehensive, literature-derived, population-based quantitative metric that evaluates individual diets by assessing the effects of dietary factors on several markers of inflammation, including interleukin (IL)-6, C-reactive protein (CRP), and tumor necrosis factor-α (TNF-α) [10-13]. Several meta-analyses of cohort studies in Western nations have revealed that a higher E-DII is associated with an increased risk of all-cause, CVD, and cancer mortality [14-16].

While dietary patterns differ between Asian and Western countries, previous studies in Asian populations have revealed that a higher E-DII is associated with elevated serum levels of high-sensitivity CRP [17,18]. Additionally, a high value for this index has been linked to increased cancer-associated mortality in a Korean cohort [19]. However, only a few prospective studies have evaluated the association of E-DII with mortality risk in Asian populations, particularly among adult Koreans in the general population. The present study aimed to determine the association between E-DII and the risk of all-cause, CVD, and cancer mortality in a Korean population using data from a population-based, prospective cohort study.

## MATERIALS AND METHODS

### Study population

**Figure 1.**
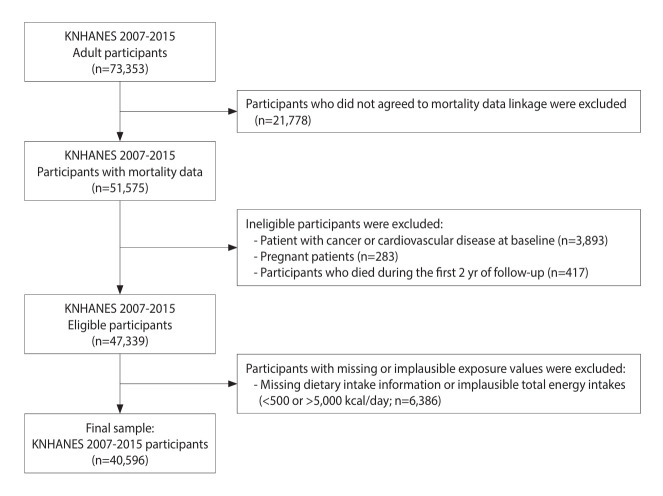
Flowchart of the study population. KNHANES, Korea National Health and Nutrition Examination Survey.

The study participants were enrolled in the Korea National Health and Nutrition Examination Survey (KNHANES) 2007-2015, a nationally representative, repeated cross-sectional survey conducted in Korea [20]. Participants were selected using a stratified multistage probability cluster sampling method based on sex, age, and region. Among the 51,575 KNHANES participants aged >19 years who consented to the mortality data linkage through December 31, 2019, individuals with cancer (n=1,803), CVD (n=2,090), or current pregnancy (n=283) at baseline were excluded. In addition, participants who died during the first 2 years of follow-up (n=417), along with those who did not complete the dietary assessments or who exhibited an implausible total energy intake (<500 or >5,000 kcal/day; n=6,386), were excluded from the analysis. Consequently, 40,596 participants were included in the present study (Figure 1).

### Mortality assessment

Mortality data were obtained by reviewing the death certificates and medical records of all eligible participants through December 31, 2019. Over a mean follow-up period of 8.2 years, 2,070 of the 40,596 individuals died. The causes of death from chronic diseases were categorized according to the International Classification of Diseases, 10th revision (ICD-10) [21]. Cause-specific mortality was coded individually for CVD (ICD-10 codes I00-I99; n=454), cancer (ICD-10 codes C00-D48; n=637), and external causes (ICD-10 codes V01-Y98; n=184).

### Determination of energy-adjusted dietary inflammatory index

The E-DII includes 45 dietary factors identified across 1,943 research studies investigating the effects of macronutrients, micronutrients, and food items on markers of inflammation, including IL-1β, IL-4, IL-6, IL-10, TNF-α, and CRP [9]. The intake of each component was assessed using a single 24-hour recall dietary survey in the KNHANES. In the present study, the KNHANES provided data for 15 food components, which were categorized as pro-inflammatory (carbohydrate, protein, total fat, and iron) or anti-inflammatory (vitamins A, B1, B2, B3, C, carotene, onion, garlic, ginger, pepper, and tea). For each of these E-DII components, z-scores were calculated by subtracting the global mean intake—derived from data from 11 countries, including Korea—from an individual’s intake and dividing the result by the global standard deviation. Each E-DII component was scored based on intake per 1,000 kcal [22]. To reduce the effect of “right skewing,” these z-scores were transformed into percentile scores by ranking the values and dividing by the total number of observations. To achieve a symmetrical distribution centered at zero and ranging from -1 (indicating maximally anti-inflammatory potential) to +1 (indicating maximally pro-inflammatory potential), the percentile scores were scaled by multiplying by 2 and subtracting 1. The final E-DII score was computed by weighting each scaled score with its corresponding inflammatory effect coefficient and summing across all dietary components [9]. A higher E-DII indicates a more pro-inflammatory diet.

### Covariates

At enrollment, all participants completed self-administered questionnaires on socio-demographic and lifestyle factors, including sex, age, geographical region, education level, household income, smoking status, alcohol intake, and physical activity. Physical activity was assessed using metabolic equivalent of task (MET)-min/wk, calculated by multiplying a value representing the intensity (2.4, 5.0, and 7.5 for light, moderate, and vigorous activity, respectively) by the average frequency of physical activity per week. All participants underwent clinical examinations, including blood tests for triglycerides, fasting glucose, and blood cholesterol levels; systolic and diastolic blood pressure measurements; and anthropometric measurements including height, weight, and waist circumference. Medical history of diseases, such as diabetes mellitus, hypertension, and dyslipidemia, was determined by synthesizing information on physician diagnosis, clinical measurements, and medication use. Body mass index (BMI) was calculated by dividing body weight (in kilograms) by the square of height (in meters).

### Statistical analysis

Continuous variables were expressed as mean (standard deviation), with differences evaluated using one-way analysis of variance. Categorical variables were presented as number (percentage), with differences compared using the chi-square test. Participants were divided into tertiles based on E-DII scores. Using Cox proportional hazards models, multivariate hazard ratios (HRs) with 95% confidence intervals (CIs) were calculated for the risk of all-cause, CVD, and cancer mortality, comparing the E-DII values of the second and third tertiles with that of the first (lowest) tertile. The multivariate models included the following potential confounders: model 1 was adjusted for age, sex, residential area, education level, occupation, smoking status, alcohol intake, physical activity (MET-min/wk), total energy intake, and obesity, while model 2 was adjusted for diabetes mellitus, dyslipidemia, and hypertension in addition to the variables in model 1. To investigate these associations, we performed Cox proportional hazards analyses for each component, adjusting for the same confounders and using a dietary inflammatory index recalculated by excluding the component of interest. The p-values represent comparisons between the third and first tertiles, with tertile 1 as the reference group.

Subgroup analyses were performed by age (<50 or ≥50 years), sex, smoking status (never, former, or current smoker), education level (less than high school, high school, or college or higher), total energy intake (below or above the median intake), BMI (<25.0 or ≥25.0 kg/m^2^), physical activity (below or above the median MET-min/wk), and medical history (diabetes mellitus, hypertension, and dyslipidemia). The significance of interaction terms was assessed using the Wald test to evaluate differences in mortality risks across subgroups. For sensitivity analysis, participants who died during the first 3 years of follow-up (n=207) and those who died from external causes (n=184), along with participants meeting other specific criteria, were excluded. A Cox proportional hazards model, including the same potential confounders, was then applied to the selected participants. All statistical analyses were performed using Stata/SE version 13.0 (StataCorp, College Station, TX, USA), and a p-value of less than 0.05 was considered to indicate statistical significance.

### Ethics statement

All participants provided informed consent, and the survey was approved by the Ethics Committee of the Korea Centers for Disease Control and Prevention (Nos. 2007-02CON-04-P, 2008-04EXP-01-C, 2009-01COM-03-2C, 2010-02CON-21-C, 2011-02CON-06-C, 2012-01EXP-01-2C, 2013-07CON-03-4C, 2013-12EXP-03-5C, and 2015-01-02-6C).

## RESULTS

### Participant characteristics

The mean E-DII at baseline was 0.0±0.3 (range, -3.1 to 2.7). Table 1 presents the baseline demographic characteristics of participants categorized by E-DII tertile. Compared with those in the lowest E-DII category (tertile 1), participants in the higher tertiles (tertiles 2 and 3) were more likely to be older female residing in rural areas and to have lower levels of education and household income. Participants with a higher E-DII were also more likely to be current smokers, to consume alcohol less frequently, and to engage in lower physical activity; furthermore, they displayed lower obesity rates, although they exhibited a higher prevalence of diabetes mellitus.

### Association of energy-adjusted dietary inflammatory index with mortality

Table 2 presents the associations of E-DII with all-cause, cancer, and CVD mortality. Using the lowest E-DII group (tertile 1) as the reference, participants in the highest tertile (tertile 3) faced significantly elevated risk of all-cause (HR, 1.52; 95% CI, 1.32 to 1.76), cancer (HR, 1.39; 95% CI, 1.08 to 1.77) and CVD (HR, 1.64; 95% CI, 1.18 to 2.29) mortality. After additional adjustment for diabetes mellitus, dyslipidemia, and hypertension, significant associations persisted for all-cause (HR, 1.45; 95% CI, 1.25 to 1.69), cancer (HR, 1.41; 95% CI, 1.09 to 1.81), and CVD (HR, 1.53; 95% CI, 1.07 to 2.18) deaths. In sensitivity analysis, associations with all-cause and CVD mortality remained significant, although significance was no longer observed for cancer mortality (Supplementary Material 1).

Table 3 presents the association between the consumption of specific E-DII food components and all-cause mortality. In summary, only higher intakes of protein, fat, vitamin B1, niacin, onion, and ginger were associated with decreased mortality after adjusting for all other E-DII components.

### Associations of energy-adjusted dietary inflammatory index with baseline characteristics after stratification

**Figure 2.**
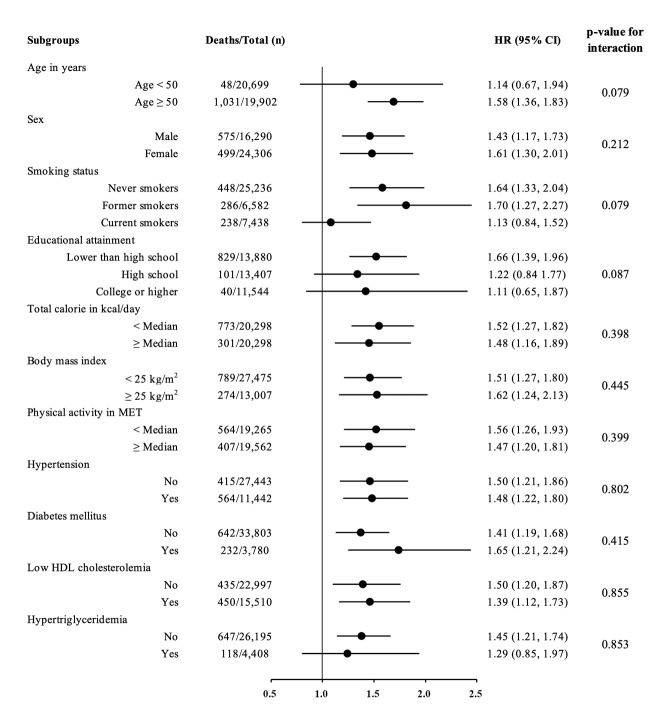
Forest plot showing the risk of all-cause mortality associated with a high energy-adjusted dietary inflammatory index based on multivariable analysis with stratification. Risk was calculated by comparing tertile 3 to tertile 1 (reference), adjusted for age, sex, education, geographic region, smoking status, physical activity measured in MET-min/wk, total caloric intake, and obesity. HR, hazard ratio; CI, confidence interval; MET, metabolic equivalent of task; HDL, high-density lipoprotein.

Figure 2 displays the associations between E-DII and all-cause mortality following stratification of the cohort by age, sex, smoking status, education level, total caloric intake, BMI, physical activity, and medical conditions, including hypertension, diabetes mellitus, low high-density lipoprotein cholesterol levels, and hypertriglyceridemia. A positive association between E-DII and mortality was observed in most subgroups, with HRs estimated by comparing the highest and lowest tertiles. Specifically, following stratification based on median age, the risk of all-cause mortality was significantly associated with E-DII in participants aged ≥50 years (HR, 1.58; 95% CI, 1.36 to 1.83), but not in those aged <50 years. Analyses stratified by sex, education level, total caloric intake, BMI, physical activity, blood pressure, and high-density lipoprotein cholesterol level revealed significant associations for all groups, with no significant differences between them. The strongest association between E-DII and all-cause mortality was observed among past smokers (HR, 1.70; 95% CI, 1.27 to 2.27), while no significant relationship was found among current smokers. Finally, the E-DII was significantly associated with all-cause mortality in participants with diabetes mellitus and in those with normal triglyceride levels (Figure 2).

## DISCUSSION

In this nationally representative sample, we found that consuming a more pro-inflammatory diet, as quantified based on E-DII, was associated with a higher risk of all-cause, CVD, and cancer mortality compared with consuming a more anti-inflammatory diet. These results suggest that increasing the intake of anti-inflammatory dietary components could reduce the risk of all-cause, cancer, and CVD mortality.

Our findings are consistent with a meta-analysis of 12 prospective studies reporting that following a pro-inflammatory diet with a higher E-DII was associated with a greater risk of all-cause mortality [23]. Another meta-analysis of 14 studies, including 2 case-control studies, 11 cohort studies, and 1 cross-sectional study, revealed that the risk of CVD incidence and mortality was 36% higher among the highest E-DII category compared with the lowest category [15]. A meta-analysis of 24 studies of patients with cancer reported that a pro-inflammatory diet with a higher E-DII could contribute to substantial increases in cancer incidence, odds, and mortality [15]. All studies in these meta-analyses used the same procedure to develop and validate the E-DII, providing strong, direct prospective evidence that inflammatory mechanisms explain the association between unhealthy diet and premature death. Our findings support the use of E-DII to capture the inflammatory potential and long-term health effects of diets in Asian countries, particularly in the Korean population. The E-DII was developed to ensure broad applicability across diverse populations by integrating actual dietary intake data from multiple countries, including Korea, and by employing a standardized approach such as z-scoring based on global dietary intake distributions. Its validity in assessing diet-induced inflammatory potential has been previously established in Asian populations, including Koreans [24,25]. In the present study, we further substantiated its validity as an inflammatory index by demonstrating a significant association between E-DII score and high-sensitivity CRP level in a subset of participants.

Multiple biological pathways underlie the consistently demonstrated association between E-DII and health effects, including mortality risk [15,16]. As a contributor to chronic inflammation [6,26,27], a pro-inflammatory diet can elevate the levels of several cytokines, such as IL-1 and TNF-α, which are involved in the chemotaxis and migration of inflammatory cells into the bloodstream [28,29]. These pro-inflammatory molecules increase the expression of cell adhesion molecules, such as selectins and cadherins, which mediate the adhesion of white blood cells to the vascular endothelium [30]. Additionally, diet plays a critical role in increasing the risk of CVD and cancer through pathways involving body fat [31]. For example, a high BMI is associated with dysregulation of several metabolic risk factors, such as high cholesterol levels, high blood pressure, and insulin resistance, which in turn are linked to increased cancer risk and poor prognosis [32,33]. The link between inflammation and disease may also influenced by shifts in microbiome composition, which contribute to immune activation and key cellular pathways including NF-κB signaling, production of pro-inflammatory cytokines, and modulation of T helper cell subsets [34-36]. Furthermore, certain dietary factors may directly or indirectly impact key epigenetic mechanisms involved in DNA repair and cell cycle regulation, such as DNA methylation, histone modifications, and chromatin remodeling [37]. For instance, green tea polyphenols have been shown to interfere with epigenetic mechanisms in early carcinogenesis in breast cancer by reversing the abnormal DNA methylation and histone acetylation that alter gene regulation in tumor cells [38].

Beyond its strong and consistent association with mortality, the relationship between high E-DII and pro-inflammatory markers has been reported in studies across various countries, including Korea [9,17]. E-DII captures the potential inflammatory impact of a diet—a factor not usually considered when assessing dietary health. The 45 dietary components used in the original E-DII include several macronutrients and micronutrients; food items such as garlic, ginger, and onion; and key bioactive polyphenols such as flavonoids [15]. In the present study, only 15 dietary components of the E-DII were available in the KNHANES database. Nonetheless, the range of E-DII scores observed here differed somewhat from those reported in previous studies, while the overall trend remained consistent. In our study, E-DII scores ranged from approximately -3.5 to 3.1, whereas prior studies have reported approximate ranges of -3.7 to 0.7 [39] or -2.0 to 2.9 [40]. Although specific cut-off values vary across studies, the general trend of higher E-DII scores being associated with increased health risks has been consistently observed. While the association of E-DII and inflammatory markers with disease has already been demonstrated in Asian populations [17,41,42], this study is notable for validating the prospective impact of this dietary index on mortality.

We also explored the independent associations of specific E-DII components with mortality. Our analyses revealed that high intakes of certain dietary components, such as protein, fat, vitamin B1, niacin, onion, and ginger, were independently associated with decreased mortality risk, even after adjusting for all remaining E-DII components. A diet rich in vitamins, onions, and ginger—characteristic of a diet high in vegetables and fruits—can reduce disease risk by improving vascular function, reducing inflammation, and supporting immune function [27,32]. As a nutrient with complex effects on inflammatory mechanisms, fat has a dichotomous impact on health. A diet high in saturated fat increases pro-inflammatory cytokine levels while reducing anti-inflammatory cytokine levels, thereby weakening the innate immune response and fostering an inflammatory environment [27]. However, in the Korean diet, a relatively high fat intake reflects a moderate fat/carbohydrate ratio, which is associated with a lower mortality risk [43]. The mechanism underlying the distinct association between total fat intake and inflammation in Asian populations remains unclear; nevertheless, in the present study, a higher E-DII score for total fat intake was associated with greater inflammatory potential.

In the present study, no other E-DII component independently drove the association between a pro-inflammatory diet and mortality risk, raising the possibility that the impact of individual E-DII components on health may be significant only in combination. Dietary assessments that capture the combined effects of multiple dietary components, rather than single nutrients or individual foods, represent a strength of the E-DII [44]. Additionally, the E-DII evaluates the associations between health outcomes and dietary patterns using a consistent method, unlike the Mediterranean or other diets, which may lack precision due to the diverse methods used to assess specific dietary patterns [45].

The HRs for mortality risk in participants with a higher E-DII remained significantly elevated even after adjusting for multiple confounding variables, including age, sex, smoking status, education level, caloric intake, BMI, physical activity, diabetes, dyslipidemia, and hypertension. Notably, stratified analyses revealed a robust prospective association between E-DII and all-cause mortality across various demographic and clinical subgroups. For example, individuals aged ≥50 years had a higher mortality risk than those aged <50 years. However, the difference between the subgroups was not significant, suggesting an absence of age-related vulnerability to dietary inflammation. Similarly, the mortality risk was higher among patients with diabetes than in those without the condition, suggesting that impaired blood glucose metabolism or insulin resistance may exacerbate the inflammatory response. In a meta-analysis of the dose-response association between dietary inflammatory potential and all-cause mortality, the risk was lower in individuals aged 60 years or older, highlighting the need for further investigation into the impact of age on the diet–mortality association. Regarding sex differences, a previous study demonstrated that the effect of E-DII on mortality was slightly more limited in female compared with male [46], although we did not observe significant sex differences in the present study. Overall, our stratified analyses underscore the importance of precisely identifying populations that would benefit most from public health interventions aimed at reducing dietary inflammation to improve overall survival.

The strengths of the present study include the use of a large, nationally representative cohort; the administration of a 24-hour dietary recall survey by trained dietitians; the utilization of validated blood sample collection and analysis protocols; and the inclusion of key lifestyle covariates. Another strength is the use of the validated E-DII to assess the inflammatory potential of food and nutrients. Diet remains a highly modifiable risk factor for multiple chronic diseases and can be targeted through health policies and public health interventions. E-DII serves as a useful summary measure of the total inflammatory potential of multiple food items and can be used to estimate the potential burden of diet-related disease in epidemiologic studies of subpopulations, thus informing disease prevention efforts. Our findings were consistent across multiple sensitivity analyses with various exclusions, including deaths from external causes, deaths during the first 3 years of the KNHANES follow-up period, and participants with diabetes mellitus at baseline, in addition to CVD and cancer.

The limitations of the present study should also be acknowledged. First, the E-DII was derived from a dietary assessment tool based on self-reporting using a 24-hour recall survey, which can lead to recall bias and misclassification of exposure. This method also does not fully capture participants’ usual intake, as it overlooks seasonal variability and individual preferences. Second, the E-DII was calculated only once at baseline, and participants’ diets might have changed during the follow-up period. However, a previous study reported that dietary patterns in adults remain relatively stable over time [47]. Third, we observed heterogeneity in the original E-DII, including differences in the number of food and nutrient parameters used for its calculation. Specifically, the 21-component E-DII was available for the KNHANES data only after 2013 and thus could not be utilized in our mortality analysis. However, we found that the 15-component E-DII used in this study correlated significantly with the 21-component version (*r*=0.857, p<0.001). Furthermore, the 15-component E-DII demonstrated a significant association with high-sensitivity CRP level, a well-established marker of systemic inflammation (Table 1). These findings suggest that the 15-component E-DII sufficiently reflects the inflammatory characteristics captured by the original E-DII, supporting its robustness despite the reduced number of components. Finally, given the relatively short follow-up duration, with a mean of 8.2 years, and the low number of deaths, we cannot completely exclude the possibility of reverse causation due to subclinical disease status. Moreover, we were unable to distinguish the specific types of CVD and cancer that led to death in our cohort. To minimize these limitations, we excluded patients with CVD and cancer at baseline and those who died during the first 2 years of the KNHANES follow-up period.

In conclusion, the observed association of the E-DII with all-cause, CVD, and cancer mortality in the Korean population suggests that an anti-inflammatory diet may help minimize the disease burden associated with all-cause death and mitigate the severity of CVD and cancer.

## Figures and Tables

**Figure f3-epih-47-e2025017:**
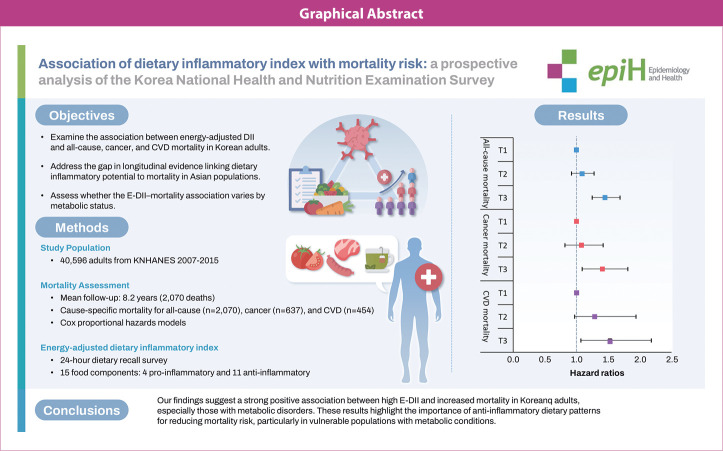


**Table 1.** Baseline characteristics of participants in the KNHANES 2007-2015 mortality follow-up study, stratified by tertiles of the E-DII

Characteristics	Tertile 1 (n=13,532)	Tertile 2 (n=13,532)	Tertile 3 (n=13,532)	p-value^1^
E-DII	−1.1±0.5	0.0±0.3	1.1±0.5	<0.001
Age (yr)	48.6±14.5	48.7±15.9	51.4±18.0	0.005
20-39	3,976 (29.6)	4,328 (32.3)	4,029 (30.2)	<0.001
40-59	6,138 (45.6)	5,301 (39.6)	4,143 (31.1)	
≥60	3,334 (24.8)	3,752 (28.0)	5,157 (38.7)	
Male	5,393 (39.9)	5,484 (40.5)	5,413 (40.0)	0.495
Residential area				
Urban	10,888 (80.5)	10,605 (78.4)	9,929 (73.4)	<0.001
Rural	2,644 (19.5)	2,927 (21.6)	3,603 (26.6)	
Education level				
Less than high school	3,812 (29.3)	4,376 (33.7)	5,692 (44.4)	<0.001
High school graduate	4,743 (36.5)	4,583 (35.3)	4,081 (31.8)	
College or higher	4,450 (34.2)	4,043 (31.1)	3,051 (23.8)	
Household income				
First quartile (lowest)	1,740 (13.0)	2,309 (17.3)	3,68 (127.7)	<0.001
Second quartile	3,243 (24.2)	3,396 (25.5)	3,590 (26.4)	
Third quartile	3,959 (29.6)	3,787 (28.4)	3,230 (24.3)	
Fourth quartile (highest)	4,441 (33.2)	3,850 (28.9)	2,874 (21.6)	
Occupation				
Non-physical labor	4,963 (38.3)	4,393 (33.9)	3,426 (26.8)	<0.001
Physical labor	3,185 (24.6)	3,371 (26.0)	3,608 (28.2)	
Unemployed, homemaker, and other	3,959 (29.6)	5,186 (40.1)	5,746 (45.0)	
Alcohol intake				
Less than once a month	4,255 (37.5)	4,242 (37.6)	4,400 (40.2)	<0.001
Once a month or more	7,082 (62.5)	7,031 (62.4)	6,516 (59.7)	
Smoking status				
Never smoker	8,679 (66.0)	8,437 (64.2)	8,120 (62.6)	<0.001
Former smoker	2,246 (17.1)	2,158 (16.4)	2,178 (16.8)	
Current smoker	2,232 (17.0)	2,540 (19.4)	2,666 (20.6)	
Physical activity (MET-min/wk)	2,187.6±3,514.5	2,065.9±3,610.6	2,026.3±3,654.3	<0.001
BMI (kg/m^2^)				
Underweight (BMI <18.5)	450 (3.3)	619 (4.6)	694 (5.2)	<0.001
Normal (18.5 ≤ BMI <25.0)	8,608 (63.7)	8,598 (63.7)	8,506 (63.1)	
Obese (BMI ≥25.0)	4,450 (32.9)	4,272 (31.7)	4,285 (31.8)	
Hypertension	3,592 (27.6)	3,373 (28.7)	4,113 (32.0)	<0.001
Diabetes mellitus	1,147 (9.1)	1,244 (9.9)	1,389 (11.3)	<0.001
Dyslipidemia	6,565 (50.5)	12,929 (48.9)	6,420 (51.0)	0.002
High-sensitivity C-reactive protein (mg/L)	1.2±2.2	1.1±1.9	1.4±2.7	0.005
Total calories (kcal/day)	2,093.1±21.6	2,037.5±809.4	1,955.6±828.9	<0.001

Values are presented as mean±standard deviation or number (%). KNHANES, Korea National Health and Nutrition Examination Survey; E-DII, energy-adjusted dietary inflammatory index; BMI, body mass index; MET, metabolic equivalent of task.

1 Using one-way analysis of variance for continuous variables and the chi-square test for categorical variables.

**Table 2.** Multivariable analysis showing the association of the E-DII with all-cause, cancer, and CVD mortality in the KNHANES 2007-2015 mortality follow-up study^1^

Variables	E-DII			p-value
	Tertile 1	Tertile 2	Tertile 3	
Range (Min, Max)	-3.49, -0.49	-0.49, 0.47	0.47, 3.13	
All-cause mortality				
Deaths/PY	444/111,481	552/111,102	1,074/110,186	
Weighted deaths/weighted PY	237,709/93,287,461	305,609/94,213,789	539,656/91,884,997	
Model 1	1.00 (reference)	1.15 (0.87, 1.35)	1.52 (1.32, 1.76)	<0.001
Model 2	1.00 (reference)	1.09 (0.92, 1.28)	1.45 (1.25, 1.69)	<0.001
Cancer mortality				
Deaths/PY	144/109,564	186/110,644	307/112,562	
Weighted deaths/weighted PY	74,892/93,586,130	103,293/93,147,996	157,907/92,652,121	
Model 1	1.00 (reference)	1.11 (0.85, 1.44)	1.39 (1.08, 1.77)	0.009
Model 2	1.00 (reference)	1.08 (0.82, 1.42)	1.41 (1.09, 1.81)	0.009
CVD mortality				
Deaths/PY	82/111,482	132/111,102	240/110,186	
Weighted deaths/weighted PY	41,458/93,287,461	64,665/94,213,789	110,622/91,884,997	
Model 1	1.00 (reference)	1.37 (0.97, 1.94)	1.64 (1.18, 2.29)	0.003
Model 2	1.00 (reference)	1.29 (0.89, 1.87)	1.53 (1.07, 2.18)	0.019

Values are presented as hazard ratio (95% confidence interval). E-DII, energy-adjusted dietary inflammatory index; CVD, cardiovascular disease; KNHANES, Korea National Health and Nutrition Examination Survey; Min, minimum; Max, maximum; PY, person-years.

1 Hazard ratios refer to a 1-tertile change in the value of the covariate; Model 1 was adjusted for age, sex, residential area, education level, occupation, smoking status, alcohol intake, physical activity measured by metabolic equivalent of task-min/wk, total energy intake, and obesity; In addition to the variables from model 1, model 2 was adjusted for diabetes mellitus, dyslipidemia, and hypertension.

**Table 3.** Multivariable analysis of the association of specific E-DII components with all-cause mortality^1^

Variables	Consumption of the specific E-DII component			p-value
	Tertile 1	Tertile 2	Tertile 3	
Carbohydrate				
Deaths/PY	361/107,469	497/111,607	1,212/113,694	
Model 1	1.00 (reference)	0.86 (0.74, 0.99)	1.10 (0.96, 1.25)	0.183
Model 2	1.00 (reference)	0.88 (0.75, 1.03)	1.12 (0.97, 1.29)	0.114
Model 3	1.00 (reference)	0.89 (0.76, 1.05)	1.12 (0.97, 1.29)	0.121
Protein				
Deaths/PY	1,023/108,290	588/110,561	459/113,918	
Model 1	1.00 (reference)	0.80 (0.71, 0.89)	0.77 (0.68, 0.87)	<0.001
Model 2	1.00 (reference)	0.79 (0.70, 0.89)	0.76 (0.66, 0.86)	<0.001
Model 3	1.00 (reference)	0.84 (0.75, 0.95)	0.83 (0.73, 0.95)	0.008
Fat				
Deaths/PY	1,266/112,646	540/111,888	264/108,236	
Model 1	1.00 (reference)	0.82 (0.73, 0.91)	0.76 (0.65, 0.88)	<0.001
Model 2	1.00 (reference)	0.81 (0.72, 0.92)	0.76 (0.65, 0.90)	0.001
Model 3	1.00 (reference)	0.84 (0.75, 0.95)	0.80 (0.68, 0.94)	0.005
Iron				
Deaths/PY	892/117,935	584/109,746	594/105,089	
Model 1	1.00 (reference)	0.88 (0.79, 0.99)	0.82 (0.73, 0.92)	0.001
Model 2	1.00 (reference)	0.88 (0.78, 1.00)	0.80 (0.71, 0.91)	0.001
Model 3	1.00 (reference)	0.96 (0.85, 1.09)	0.92 (0.81, 1.05)	0.209
Vitamin A				
Deaths/PY	1,029/106,789	520/110,301	521/115,680	
Model 1	1.00 (reference)	0.76 (0.68, 0.86)	0.72 (0.64, 0.81)	<0.001
Model 2	1.00 (reference)	0.79 (0.70, 0.89)	0.74 (0.66, 0.84)	<0.001
Model 3	1.00 (reference)	0.88 (0.78, 1.00)	0.92 (0.78, 1.08)	0.302
Vitamin B1				
Deaths/PY	1,079/125,708	587/115,025	404/92,037	
Model 1	1.00 (reference)	0.82 (0.73, 0.91)	0.84 (0.74, 0.95)	0.005
Model 2	1.00 (reference)	0.83 (0.74, 0.93)	0.77 (0.67, 0.89)	<0.001
Model 3	1.00 (reference)	0.88 (0.79, 0.99)	0.84 (0.73, 0.97)	0.018
Vitamin B2				
Deaths/PY	1,155/113,274	497/112,116	418/107,380	
Model 1	1.00 (reference)	0.78 (0.70, 0.86)	0.84 (0.75, 0.95)	0.007
Model 2	1.00 (reference)	0.80 (0.71, 0.91)	0.85 (0.74, 0.97)	0.013
Model 3	1.00 (reference)	0.90 (0.79, 1.01)	0.99 (0.86, 1.15)	0.944
Niacin				
Death/PY	976/108,613	642/112,557	452/111,600	
Model 1	1.00 (reference)	0.82 (0.74, 0.92)	0.75 (0.66, 0.84)	<0.001
Model 2	1.00 (reference)	0.84 (0.75, 0.95)	0.75 (0.66, 0.85)	<0.001
Model 3	1.00 (reference)	0.90 (0.80, 1.02)	0.83 (0.73, 0.95)	0.005
Carotene				
Deaths/PY	978/106,020	531/110,545	561/116,205	
Model 1	1.00 (reference)	0.73 (0.65, 0.82)	0.71 (0.63, 0.79)	<0.001
Model 2	1.00 (reference)	0.73 (0.66, 0.84)	0.74 (0.65, 0.83)	<0.001
Model 3	1.00 (reference)	0.82 (0.72, 0.94)	0.92 (0.79, 1.08)	0.307
Vitamin C				
Death/PY	829/103,425	678/116,944	563/112,401	
Model 1	1.00 (reference)	0.80 (0.72, 0.89)	0.76 (0.68, 0.85)	<0.001
Model 2	1.00 (reference)	0.81 (0.72, 0.91)	0.78 (0.69, 0.88)	<0.001
Model 3	1.00 (reference)	0.88 (0.78, 0.99)	0.89 (0.78, 1.02)	0.098
Onion				
Death/PY	1,161/121,429	464/104,324	445/107,017	
Model 1	1.00 (reference)	0.77 (0.68, 0.87)	0.76 (0.68, 0.86)	<0.001
Model 2	1.00 (reference)	0.77 (0.67, 0.87)	0.76 (0.67, 0.87)	<0.001
Model 3	1.00 (reference)	0.80 (0.70, 0.91)	0.82 (0.72, 0.94)	0.003
Garlic				
Deaths/PY	904/116,069	608/107,178	558/109,524	
Model 1	1.00 (reference)	0.96 (0.86, 1.07)	0.88 (0.78, 0.98)	0.025
Model 2	1.00 (reference)	0.92 (0.82, 1.04)	0.87 (0.77, 0.98)	0.021
Model 3	1.00 (reference)	0.99 (0.87, 1.11)	0.99 (0.87, 1.13)	0.888
Ginger				
Deaths/PY	1,780/254,865	152/38,132	138/39,775	
Model 1	1.00 (reference)	0.81 (0.67, 0.97)	0.78 (0.65, 0.94)	0.011
Model 2	1.00 (reference)	0.79 (0.65, 0.96)	0.78 (0.64, 0.94)	0.011
Model 3	1.00 (reference)	0.83 (0.69, 1.01)	0.87 (0.71, 1.06)	0.158
Tea				
Deaths/PY	2,028/314,019	19/9,497	23/9,256	
Model 1	1.00 (reference)	0.75 (0.48, 1.18)	0.99 (0.65, 1.51)	0.971
Model 2	1.00 (reference)	0.80 (0.50, 1.26)	1.05 (0.69, 1.61)	0.816
Model 3	1.00 (reference)	0.85 (0.54, 1.34)	1.13 (0.74, 1.74)	0.564
Black pepper				
Deaths/PY	1,649/210,991	204/59,698	217/62,081	
Model 1	1.00 (reference)	0.89 (0.76, 1.04)	0.83 (0.71, 0.97)	0.022
Model 2	1.00 (reference)	0.89 (0.76, 1.06)	0.84 (0.71, 0.99)	0.046
Model 3	1.00 (reference)	0.98 (0.83, 1.16)	0.93 (0.78, 1.10)	0.394

Values are presented as hazard ratio (95% confidence interval). E-DII, energy-adjusted dietary inflammatory index; PY, person-years.

1 Hazard ratios refer to a 1-quartile change in the value of the covariate; Model 1 was adjusted for age, sex, residential area, education level, occupation, smoking status, alcohol intake, physical activity measured by metabolic equivalent of task-min/wk, total energy intake, and obesity; In addition to the variables from model 1, model 2 was adjusted for diabetes mellitus, dyslipidemia, and hypertension; Model 3 included the variables from model 2 and was further adjusted for the E-DII, which was recalculated without the component of interest.

## Supplementary materials

Supplementary Material 1. Multivariable hazard ratios and 95% confidence intervals for the associations between energy-adjusted dietary inflammatory index and all-cause, cancer and cardiovascular disease mortality in the KNHANES 2007–2015 mortality follow-up study
