# Supplementary material for: Association of dietary inflammatory index with mortality risk: a prospective analysis of the Korea National Health and Nutrition Examination Survey
Source: Epidemiol Health. 2025 Apr 9;47:e2025017. doi: 10.4178/epih.e2025017 (PMC12425700; doi:10.4178/epih.e2025017)
Supplement: Supplementary Material 1. — Multivariable hazard ratios and 95% confidence intervals for the associations between energy-adjusted dietary inflammatory index and all-cause, cancer and cardiovascular disease mortality in the KNHANES 2007–2015 mortality follow-up study [file epih-47-e2025017-Supplementary-1.docx]

**Supplementary Material**

Supplementary Material 1. Multivariable hazard ratios and 95% confidence intervals for the associations between energy-adjusted dietary inflammatory index and all-cause, cancer and cardiovascular disease mortality in the KNHANES 2007–2015 mortality follow-up study

|  | E-DII tertile | | | | p value |
| --- | --- | --- | --- | --- | --- |
|  | | T1  (n = 12,168) | T2  (n = 12,168) | T3  (n = 12,169) |  |
| **All-cause mortality** | |  |  |  |  |
| Death/person-year | | 276/100,868 | 336/100,688 | 700/111,823 |  |
| Weighted death  /weighted person-year | | 140,790/85,674,980 | 179,303/86,882,595 | 336,479/85,567,214 |  |
| Model 1 | | 1.00 (ref) | 1.14 (0.92, 1.41) | 1.56 (1.29, 1.89) | <0.001 |
| Model 2 | | 1.00 (ref) | 1.10 (0.88, 1.38) | 1.49 (1.22, 1.83) | <0.001 |
| **Cancer mortality** | |  |  |  |  |
| Death/person-year | | 118/100,868 | 129/100,688 | 204/111,823 |  |
| Weighted death  /weighted person-year | | 62,188/85,674,980 | 72,936/86,882,595 | 102,257/85,567,214 |  |
| Model 1 | | 1.00 (ref) | 1.03 (0.75, 1.40) | 1.25 (0.93, 1.67) | 0.137 |
| Model 2 | | 1.00 (ref) | 1.00 (0.72, 1.39) | 1.29 (0.95, 1.75) | 0.100 |
| **CVD mortality** | |  |  |  |  |
| Death/person-year | | 56/100,868 | 87/100,688 | 171/111,823 |  |
| Weighted death  /weighted person-year | | 28,678/85,674,980 | 42,460/86,882,595 | 81,000/85,567,214 |  |
| Model 1 | | 1.00 (ref) | 1.34 (0.85, 2.11) | 1.80 (1.18, 2.74) | 0.006 |
| Model 2 | | 1.00 (ref) | 1.29 (0.80, 2.07) | 1.65 (1.05, 2.57) | 0.029 |

Hazard ratios refer to a one-tertile change in the value of the covariate. Model 1 was adjusted for age, sex, residential area, education level, occupation, smoking status, alcohol intake, physical activity by metabolic equivalent of task, total energy intake, and obesity. Model 2 was adjusted using the variables in Model 1 in addition to diabetes mellitus, dyslipidemia, and hypertension.

CVD, cardiovascular disease; E-DII, energy-adjusted dietary inflammatory index
